# Frailty as an integrative marker of physiological vulnerability in the era of COVID-19

**DOI:** 10.1186/s12916-020-01809-1

**Published:** 2020-10-23

**Authors:** Qian-Li Xue

**Affiliations:** 1 Department of Medicine Division of Geriatric Medicine and Gerontology, School of Medicine, Johns Hopkins University, Baltimore, MD USA; 2 Center on Aging and Health, Johns Hopkins Medical Institutions, Baltimore, MD USA

**Keywords:** Aging, Geriatric syndrome, Reserve, Resilience

## Background

As researchers across the globe race to develop vaccines and treatments for COVID-19, evidence is mounting that disease severity, prognosis, and outcomes among persons infected with SARS-CoV-2 are vastly heterogeneous. Based on early studies, age and preexisting disease burden are increasingly being used as risk stratifiers in clinical decision-making when treating COVID-19 patients. However, a question worth asking is whether rushed, few-sizes-fit-all decision-making in a crisis as life-altering as the COVID-19 pandemic will cause unintended harms with respect to access to and delivery of care. In this regard, the observational study conducted by Ma et al. in a sample of older patients with confirmed COVID-19 pneumonia in Wuhan China provided initial evidence that frailty, a clinical syndrome of systemic vulnerability, may be a powerful predictor of disease prognosis and outcomes [1]. It is the finding that the predictive relationship was independent of comorbidity and chronological age that makes the study most interesting. This suggests that the concept of frailty captures important information regarding underlying vulnerability that age and comorbidity together do not fully explain.

## Main text

In theory, frailty is conceptualized as a state of decreased ability for multiple physiologic systems to interact harmoniously in order to maintain physiologic equilibrium [2]. This compromised state of health affects the normal complex adaptive behavior that is essential to resilient stress response. The FRAIL scale used by Ma et al. was modeled after the physical frailty phenotype composed of mutually exacerbating clinical manifestations (a.k.a., the “cycle of frailty” [2]) that includes skeletal muscle decline, lower levels of energy production, and altered nutritional intake and processing. Even though disability, comorbidity, and/or frailty often coexist in older adults, there is increasing consensus that frailty is a unique clinical entity different from disability and comorbidity. Independent of Ma et al.’s finding, evidence in support of this theory is mounting that disability, comorbidity, and age separately or in combination do not fully explain substantial differences in health outcomes across patient populations (e.g., geriatric vs. disease-specific populations) and clinical settings (e.g., primary care vs. elective surgery) [3]. Therefore, the concept of frailty provides a new window into latent vulnerability that is both integrative and systemic rather than system- or organ-specific.

While our knowledge on the pathophysiology of COVID-19 is evolving, there are notable similarities in the underlying biology between COVID-19 and frailty. For example, the angiotensin-converting enzyme-2 (ACE2), as a component of the renin-angiotensin system (RAS), is a functional receptor for viral entry into host cells [4]. In addition, it is clear that those who are at highest risk of needing ventilator support and of mortality are those who experience a “cytokine storm,” i.e., an excessive production of inflammatory cytokines resulting from an increase of angiotensin II (Ang II) due to viral suppression of ACE2 expression. RAS is also hypothesized to play an important role in the pathogenesis of frailty through its aging-associated shift in the balance between the proinflammatory vs. anti-inflammatory action of Ang II type 1 receptor and Ang II type 2 receptor, respectively, resulting in increased inflammation, oxidative stress, and apoptosis [5]. Consistent with this hypothesis, studies of both humans and animals have generated evidence linking frailty to low-grade chronic inflammation (i.e., “Inflamm-aging”) [6]. In addition, a recent study in mice found that chronic treatment with the ACE inhibitor attenuated the development of frailty [7]. It is therefore conceivable that frailty may have a priming effect in the sense of manifesting a propensity of overreacting to viral infection in the form of a cytokine storm in the case of COVID-19.

The findings from Ma et al.’s study have multiple implications. First, given that Frail infected patients are at increased risk for poor outcomes, screening for frailty should be incorporated into the clinical routine at admission of infected patients in order to optimize resource allocation and clinical care. Second, treatment decisions should be made to minimize the invasiveness of medical procedures prescribed for frail patients. For example, the debate of whether the COVID-19 patients with respiratory failure should be intubated and ventilated early in the disease course in order to minimize the risk of transmission from patients to healthcare workers is also relevant to the benefit-harm assessment for the most vulnerable patients. This is because frail individuals are more susceptible to complications of invasive procedures due to their diminished ability to withstand stressors and recover from them. Therefore, less invasive treatment options such as continuous positive airway pressure should be considered. Adding to the risk of invasive procedures is hospitalization that may become more detrimental than the illness itself, particularly in a strained health care system [8]. Although balancing the different tradeoffs is no easy task, identifying frail patients based on objective data is a necessary first step to facilitate a shared decision-making between patients/family members and medical professionals.

## Implications beyond COVID-19 patients

It is important to note that the implication of the study’s findings extends beyond the infected patients. The coronavirus pandemic is affecting the lives of both the infected and the non-infected. As medical attention focuses on treating infected patients and protecting others from infection, how do we best care for frail people with the non-COVID-related disease? The radical transformation of the health care system in response to the pandemic, e.g., the replacement of in-person visit with telemedicine in order to preserve critical care capacity, has already affected our ability to maintain high-quality care for vulnerable patient populations [9]. For instance, suspending non-urgent aspects of care may not be consequential for a non-infected and non-frail patient, but it may have graver consequences for a frail patient. In a recent commentary [10], the author cited, “One of the yet-to-be-told stories of the Covid-19 pandemic is the recognition that the (necessary) proscriptions on the performance of less urgent cases has led to collateral damage to so many patients with medical conditions that truly couldn’t wait.” Rather than a broad moratorium or cancelation on elective or “routine” procedures, we need a more tailored and patient-centered approach. As modern medicine has faced unprecedented degree of uncertainty, there is an urgent need for a comprehensive risk-benefit analysis to help inform treatment decision-making during the pandemic. In this sense, the findings of this study offer new hope for increased precision in identifying high-risk population subsets, for whom preventative interventions should be the highest priority regardless of the availability and effectiveness of treatment for COVID-19.

## Data Availability

Not applicable.
